# Peer2ref: a peer-reviewer finding web tool that uses author disambiguation

**DOI:** 10.1186/1756-0381-5-14

**Published:** 2012-09-07

**Authors:** Miguel A Andrade-Navarro, Gareth A Palidwor, Carol Perez-Iratxeta

**Affiliations:** 1 Max Delbrück Center for Molecular Medicine, Robert-Rössle-Str. 10, 13125, Berlin, Germany; 2 Ottawa Hospital Research Institute, 501 Smyth Road, Ottawa, Ontario, K1H 8L6, Canada

**Keywords:** Publishing, Information storage and retrieval, MEDLINE, Peer review, Research, Natural language processing

## Abstract

**Background:**

Reviewer and editor selection for peer review is getting harder for authors and publishers due to the specialization onto narrower areas of research carried by the progressive growth of the body of knowledge. Examination of the literature facilitates finding appropriate reviewers but is time consuming and complicated by author name ambiguities.

**Results:**

We have developed a method called peer2ref to support authors and editors in selecting suitable reviewers for scientific manuscripts. Peer2ref works from a text input, usually the abstract of the manuscript, from which important concepts are extracted as keywords using a fuzzy binary relations approach. The keywords are searched on indexed profiles of words constructed from the bibliography attributed to authors in MEDLINE. The names of these scientists have been previously disambiguated by coauthors identified across the whole MEDLINE. The methods have been implemented in a web server that automatically suggests experts for peer-review among scientists that have authored manuscripts published during the last decade in more than 3,800 journals indexed in MEDLINE.

**Conclusion:**

peer2ref web server is publicly available at http://www.ogic.ca/projects/peer2ref/.

## Background

Effective expert selection for anonymous peer review is a critical step in the process of publishing research in referred scientific journals, which remains the most important platform for the dissemination of scientific knowledge. Demand for peer review is increasing as the number of researchers, journals and publications increases. In the field of biomedicine alone, the estimated number of titles currently published stands between 13,000 and 14,000, of which 5,300 are indexed in the MEDLINE database (Journals database at the NCBI, [1]).

Upon successful submission of a manuscript for publication in a journal, editors attempt to quickly identify suitable reviewers, sometimes with the assistance of authors who may have been prompted to provide several suggestions. This procedure demands a fair knowledge of the experts in the manuscript’s area of knowledge from both authors and editors. Inevitably, authors and editors will concentrate their requests on a small set of referees, usually senior authors that they know and trust. As a result, senior authors are overloaded with demands.

The bibliography offers a resource to find experts. However, the increase in the rate of production of new research makes it increasingly difficult to track all the publications coming out from even narrow fields of research and many authors that could potentially be good reviewers may not be requested. An approach to ease this problem is the development of computational methods to assist authors and editors in reviewer selection based on the literature. Such methods have the potential to facilitate the task and should produce less biased and more systematic expert selections than manual protocols.

Ideally, we would like the computer to point to potential reviewers for a given manuscript using just the manuscript content as input. One straightforward strategy in this direction is to search the database of (peer-reviewed) scientific literature for the most similar documents to the manuscript we want to review, and then suggest the authors of these documents as experts. A widely used measure of document similarity is the cosine between the abstracts of the documents encoded as vectors of the frequency of words they contain [2]. For example, BioMed Central editorial uses this approach by proposing to the associate editor that is handling the submission, the cosine values between the abstract of the submitted manuscript and abstracts referenced in MEDLINE.

There are two web server applications to find similar abstracts in MEDLINE. An early one is eTBlast [3], which uses the same principle with a more elaborated measure of text similarity that takes into account word frequencies and word order in the text. A more recent one is Jane [4], a straight implementation of Lucene's [5] MoreLikeThis algorithm, which does not take into account the words' order but their relevance according to their frequencies in a whole corpus.

Here we propose a more comprehensive approach to computational selection of peer reviewers, which relies on comparison of a word profile of the manuscript not to that of other single manuscripts but to the collection of manuscripts authored by each potential peer reviewer. This approach necessitates building word profiles for authors. However, the problem that considerably complicates the matter of identifying the manuscripts authored by one individual, especially in an automatic way, is that many authors share last names and initials with other authors. Particularities of names across countries further complicate the issue. For example, most Chinese last names are extremely common, with the eleven most common being shared by about 40% of the Chinese population, yet their wide variety of given names is lost by Western abbreviation practices [6]. Ambiguity due to given name abbreviation is a problem that affects other Asian scientists as well [7,8] in a manner well beyond the matter discussed here [9].

Dealing with author name ambiguity remains a hard problem. For the biomedical community, an obvious and ideal solution would be to have each author assigned a unique identifier in MEDLINE upon their first publication. However, this solution has no trivial implementation, as it would require the combined effort from a coordinator organization, such as the NCBI, and the whole body of the scientific community. Even if implemented today, this would not resolve the name ambiguity of the large body of prior literature.

Meanwhile, the problem is only worsening due to the ever-increasing number of scientists. Computational efforts, mostly industry-led, are being made by implementing algorithms that, by parsing MEDLINE, would partially address this matter. Some initiatives combine registration of users with profile generation and their degree of integration with companies and accessibility to their methods and data are very heterogeneous. ResearcherID from Thomson Reuters (http://www.researcherid.com/) is a company resource linked to other Thomson databases such as ISI Web of Knowledge. BioMed Experts from Collexis (http://www.biomedexperts.com/) contains a collection of automatically generated profiles for authors in MEDLINE based, according to their web site, on the concepts associated to the identifiers and on co-authors. Users can register and modify the profiles. Author-ity is an academic effort to generate author profiles [10] and offers a database of disambiguated author names in MEDLINE for download and a web interface to query it (http://arrowsmith.psych.uic.edu/arrowsmith_uic/author2.html).

Among the strategies to disambiguate authors that share the same name are the use of keywords that identify a particular subject of research, collaborators co-signing publications with the authors (networks of collaborators), physical location extracted from the affiliation data usually complemented with the years of publication, journal subject class (e.g. journals in the area of cardiology), and even co-citations in web pages [11].

Here, we have chosen to implement an approach based on co-authorship because it is straightforward, and in principle can be easily applied in an unbiased manner to every single name. Accordingly, we have attempted to disambiguate every author name in MEDLINE by co-authors and assigned different identifiers to each disambiguated instance. Next, for each identifier we derived profiles of keywords extracted from the abstracts of the references in MEDLINE associated to it. Given a manuscript, our method uses these profiles to suggest peer-reviewers based on the similarity to the keyword profile deduced from the manuscript.

## Methods

### Author name disambiguation

A major complication to identifying referees for peer review in an automatic manner is that many MEDLINE authors share both last name and initials. To address this hurdle in our expert finding tool, we have implemented a straightforward algorithm to disambiguate MEDLINE author names using coauthor names. The procedure was applied to every name in MEDLINE, defined as “last name_initials” in MEDLINE’s field [Author] and hereafter called simply “name”, and produced disambiguated profiles that consist of the set of references attributed to an author identifier. The algorithm is as follows. Given a name, we gather all MEDLINE records with that name in the author list and look for the most frequent coauthor name in the set. The subset of references with both the name of the author and most frequent coauthor is assigned to an identifier and constitutes a disambiguated author’s profile. Next, we do the same for the next most frequent coauthor name in the remaining subset of references. This procedure continues until we do not find more name repetitions among coauthors. The remaining references are singletons with different identifiers assigned to them. The application of this algorithm to MEDLINE’s 2010 release of references authored by more than one scientist resulted in 11,394,787 disambiguated authors. For a number of disambiguated authors we created a profile that describes their expertise by extracting all nouns from the abstracts of their associated references. We did this for those with at least an associated manuscript as first or last author published from 2000 to 2010 (MEDLINE release 2010) in one of more than 3,800 journals indexed in MEDLINE. The complete list of journals is available in the peer2ref website. As a result we obtained a set of 2,660,235 profiles, expectedly experts on a broad collection of subjects that covers most biological and biomedical research.

### Keyword computation from abstracts

Given an abstract we assign to each of its words a keyword score that reflects its importance in the text. To do so we consider the relationships between all words in the text as deduced from a simple model of fuzzy binary relations [12], which is based on co-occurrence within sentences. Keywords tend to be frequent words that have more and stronger relations with other words. Details of the computation can be found in [13]. The more and stronger relations a word has, the higher its keyword score will be. Computations are done over the nouns, adjectives and verbs, after filtering out stop words since this worked better than using only nouns in our benchmark (data not shown). Parts-of-speech and sentences are detected using a standard grammatical tagger (TreeTagger; Helmut Schmid, IMS, Stuttgart University http://www.ims.uni-stuttgart.de/projekte/corplex/TreeTagger/).

## Results

### Evaluation of the use of disambiguated author word profiles for referee selection

We propose that the adequacy of a disambiguated author as referee of a given manuscript can be measured in terms of the similarity between their word profile and that of the abstract of the query manuscript. To test this we designed the following benchmark. We assumed that authors from the list of references cited in a manuscript could in principle be good referees for it. Accordingly we selected 40 of the latest (at that date) published papers in PubMed Central, for which the full articles are freely available. For each paper, we ran peer2ref on title + abstract text and we checked manually whether the top suggested expert was an author of any of the references cited in the manuscript. In 8 cases, the expert was an author of the test publication itself. In these cases we skipped it and used the most up top suggestion that was not an author. The results show that in 12 cases the first suggestion is a cited author. These author profiles have an average of 16 papers and an average score of 0.54. Interestingly, the average score for authors that were not cited in the references was lower, 0.36, with a similar number of average papers per profile (16.5). One feature of our web tool (see next paragraph) is that it allows users to identify one or multiple subjects of the manuscript. This option narrows the disambiguated profiles to those that have published in journals associated to the selected subjects. When we carried out the same benchmark with subject selection, the results improved very modestly: in 15 cases the first suggestion was a cited author (with a lower average score since the authors associated to a subject are a subset of the disambiguated profiles).

### Comparison to eTBlast and Jane

To compare peer2ref with the two other available similar tools, eTBlast and Jane, we ran the same benchmark on them. The results showed that the three tools perform similarly in this task. The top suggestion was identified as a cited author in exactly 15 cases for Jane and 14 for eTblast. Interestingly, in almost as many cases (14), none of the three tools identified a cited author as a first suggestion. This indicates that some abstracts could be difficult for any tool due to a variety of reasons, like being less well written, dealing with narrow subjects of research or, critically for this particular benchmark, the corresponding manuscript having a shorter reference list (with an average of 23.8 references for these 14 cases compared to an average of 40.8 references for the rest of cases). We also noted that the overlap between the results of the three tools was relatively low. In 3 cases, cited authors were detected by the three methods, and in 11 cases cited authors were detected with only one of the methods (detected only by Jane: 2 cases; only by eTBlast: 3 cases and only by peer2ref: 6 cases). We think that this indicate that the tools are somewhat complementary. The full benchmark results are in Additional file 1: Table S1.

### Web tool implementation

Our algorithm has been implemented as the web tool peer2ref. To run peer2ref users have to paste some text from their manuscript in the input page from which at most 50 keywords with scores higher than 0.05 are used to build the profile of the manuscript. Usually a well written abstract is enough to properly reflect the content of a manuscript. However, an unstructured or too short abstract will not provide the necessary keywords for a successful search. In these cases, as well as when the research subject is highly original, additional input from the main text may be helpful (see additional information in the web tool's Supplement section).

Users can optionally select the broad subject(s) of the manuscript. Appropriate reviewers will be selected among authors that have published in the journals associated with these subjects. This allows to target referees in the particular context of a narrower subject of interest, which may not be selected in a global context due to slightly lower scores. For example, a word such as “heart” would become less discriminating in the context of the subject “Cardiology” than in a global context. Subject selection may also be handy when looking for referees for multidisciplinary research, for example, when it is necessary to summon the expertise of both geneticists and computational biologists. Classifying journals by subject is not a trivial problem. Here we are using a ready-made classification from the US National Library of Medicine which consists of 123 broad subjects.

**Figure 1:**
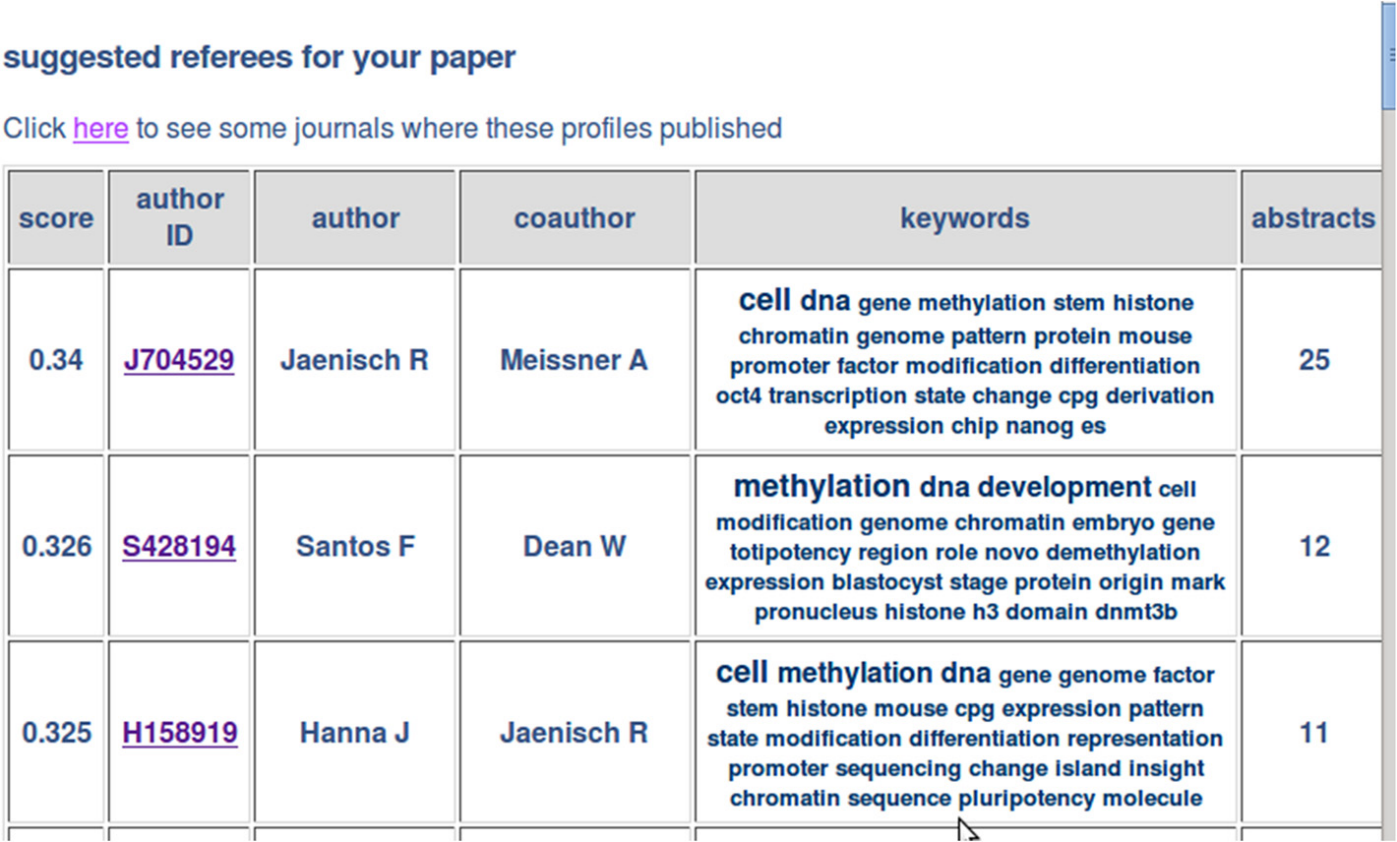
**Snapshot of the results obtained by peer2ref with reference PMID: 21468033.** Potential experts for peer-review are presented in a table with top suggestions on top, one row per profile. The included tabs are the expert's score (**score**), name (**author**) and the name coauthor (**coauthor**) that was used to disambiguate the profile, which could be used as an alternative suggestion, as well as a keyword profile (**keywords**) based on the abstracts published by the disambiguated author. A link to MEDLINE (**author ID**) is provided to quickly inspect these references.

Apache Lucene version 2.9.4, a Java based indexing and search library, was used to index the disambiguated author profiles for rapid searching. The resultant index is large, approximately half the size of the source data, however searches using the index take only seconds. The authors with the closest profiles to the query manuscript profile, according to the Lucene's *Similarity* method [5], are selected as potentially suitable referees, ranked by Lucene's scores and displayed in the results page, together with the most relevant keywords from their profiles (Figure 1).

PubMed links to the references associated with each profile allow a quick assessment of the suitability of the suggested candidates. The built-in search function of web browsers can be used to quickly find the highest ranked reviewers associated with a particular keyword. A table of journals where the resulting reviewers have published is linked from the results page. These may be considered suggestions of appropriate journals for submission of the query manuscript and can as well be used to filter the suggested profiles. In the Supplement section of the peer2ref website we have included a step by step example as well as further details and usage tips.

## Discussion

Finding experts for anonymous peer review is becoming harder due to progressive specialization of scientists on narrow research fields and other issues, such as author name ambiguity. Computational methods can facilitate this task and additionally, introduce an element of fairness into the process of referee selection as the same objective test is applied to every author to determine their adequacy as reviewer.

We have developed a fully automatic method to provide reviewer suggestions for manuscripts using a small amount of representative text, expectedly their abstracts. To address the problem of author name ambiguity, our method uses a pre-computed dataset of disambiguated authors deduced automatically from all references in MEDLINE.

To evaluate our method we carried out a manual benchmark that examined whether the top suggested reviewer for 40 abstracts was also the author of a reference cited in the corresponding manuscript. Selecting experts among the authors of the cited references of a manuscript could be a reasonable approach to find peer-reviewers manually. However, reference lists are subjective and could have biases towards particular authors and many citations could be only casually related to the subject. In the particular context of our benchmark, references lists have some disadvantages: the set of references is not a ranked list (sometimes the top suggested author was not a cited author, but the second and/or third suggestions were), and they have variable sizes, which confounds case comparison, becauseÂ in general,Â any method will perform better on average with papers with manyÂ references. Despite that, we think that this test could be used to compare peer2ref, eTBlast and Jane, and that it is in principle indicative of good performance. The results show that the three tools perform similarly.

Interestingly, in many cases the top suggested author was not found among the cited references. In principle, we do not have reasons to believe that the proposed reviewers are worse in all these cases, although on average their scores are lower. Indeed, this could be indicative that using the references as a source of reviewers may not be always an optimal strategy. Since authors will tend to cite what they know, it could be recommended to get referees that are actually not cited by the authors. Another possibility is that these type of methods are not very accurate.

As a limitation of our method, as peer2ref unique source of information is MEDLINE, it could not work well with manuscripts from fields that can be considered narrow in the context of biomedical research (e.g. engineering areas). We are aware that, because the way it was produced, i.e. solely using coauthors, our database of disambiguated authors is highly atomized, meaning that, in general, several author profiles will correspond to the same scientist, giving a high precision-low recall disambiguation. However, given its intended purpose of finding experts in narrow areas of research, this feature of our algorithm is advantageous. Often, scientists have different sets of collaborators with whom they work on somewhat different matters. This results in authors publishing manuscripts in the same small area of research with the same group of coauthors. Our disambiguated profiles partially capture this effect.

To summarize, we have developed a fully automatic method to provide suggestions of reviewers for scientific manuscripts, using text from the abstract of the manuscript and disambiguated authors profiles deduced automatically from the whole MEDLINE. We have implemented it in a public web server to make it accessible to authors and editors. We plan to update the disambiguated author profiles yearly, with each release of MEDLINE. In the future, we plan to further develop the computation of similarity scores and to add further functionality to the web tool. Peer2ref is available for public use at http://www.ogic.ca/projects/peer2ref/.

## Competing interests

The authors declare that they have no competing interests.

## Authors' contributions

MAAN and CPI conceived the method and developed it. CPI produced the disambiguation and programmed the web server. GAP implemented the Lucene indexing and searching. All authors contributed to the manuscript.

## Supplementary Material

Additional file 1 **Table S1. case**: case number; **PMIC**: PubMed Central ID; **cites**: number of citations in the reference list; **web run date**: cases runing date; **Top author peer2ref**: top suggested author by peer2ref run with no options; **score peer2ref**: score for top suggested author with no options; **abstract peer2ref**: number of abstracts associated to the disambiguated profile run with no options; **Ref**: number of references coauthored by top suggested author with no options; **topic(s)**: selection of subjects; **Top author peer2ref topic(s)**: top suggested author by peer2ref run with selection of subjects; **score peer2ref topic(s)**: score for top suggested author run with selection of subjects, a number between brackets indicates that the ranking of this profile because the top suggestion(s) was an author or the case paper; **abstract peer2ref topic(s)**: number of abstracts associated to the disambiguated profile run with selection of subjects; **Ref topic(s)**: number of references coauthored run with selection of subjects; **Top author eTBLAST**: top suggested author by eTBLAST; **score eTBLAST**: score for top suggestion by eTBLAST; **flag eTBLAST**: top suggestion is flagged as inactive; **Ref eTBLAST**: number of references coauthored by top suggestion; **detected eTBLAST:** 1 if the author was in a cited reference, 0 otherwise; **Year 1**^**st**^**hit eTBLAST**: oldest reference authored by top suggestion; **Top author Jane**: top suggested author by Jane; **confidence**: confidence score for top suggestion by Jane; **Ref Jane**: number of references coauthored by top suggestion; **detected Jane:** 1 if the author was in a cited reference, 0 otherwise; **Year 1**^**st**^**hit Jane**: oldest reference authored by top suggestion; **detected P2R (P2R topics, eTBLAST, Jane):** 1 if the top suggested author by peer2ref (peer2ref topics, eTBLAST, Jane) was in a cited reference, 0 otherwise; **Detected:** Methods for which the top suggested author was detected in the references. Click here for file
